# Evidence-based lifestyle guidance can complement radiological protection - reducing radiation-related cancer risk through lifestyle modification

**DOI:** 10.1007/s00411-026-01243-6

**Published:** 2026-08-12

**Authors:** Andrzej Wojcik, Prabodha Kumar Meher, Richard Wakeford

**Affiliations:** 1 Centre for Radiation Protection Research, Department of Molecular Biosciences, the Wenner-Gren Institute, Stockholm University, Svante Arrhenius väg 20C, 10691 Stockholm, Sweden; 2 Department of Medical Biology, Institute of Biology, Jan Kochanowski University, Kielce, Poland; 3 Centre for Occupational and Environmental Health, The University of Manchester, Manchester, UK

**Keywords:** ionising radiation, cancer reduction, cancer prevention, lifestyle

## Abstract

Radiological protection primarily manages exposure, yet public concern often focuses on health outcomes after exposure has occurred. This can foster fatalistic interpretations that cancer risk becomes fixed once a dose is received, contributing to anxiety and impaired well-being. Here we argue that such fatalism is not warranted: cancer risk is inherently conditional and depends on multiple determinants operating over time. Building on contemporary concepts of multistage carcinogenesis, radiation can contribute to initiation and may also influence promotion/progression through tissue responses; therefore, post-exposure conditions that affect inflammation, metabolism, hormonal milieu, and immune surveillance can plausibly modulate the probability that initiated lesions progress to clinically manifest cancer. We review epidemiological evidence - most notably for lung cancer - showing that lifestyle factors such as smoking interact with radiation-related risk, and we summarize additional human observations suggesting modulation by diet and infection status in the Life Span Study cohort of Japanese atomic bomb survivors. Controlled animal studies further support that obesity, dietary composition, caloric restriction, and physical activity can modify radiation-related cancer outcomes. We propose that evidence-based lifestyle guidance (e.g. smoking cessation, healthy weight, physical activity, alcohol moderation and supportive follow-up) should be communicated as an empowering complement to - never a substitute for - existing protection standards. Ethical implementation requires non-stigmatizing messaging and institutional support to ensure feasibility and equity.

## Introduction

According to the 2007 Recommendations of the International Commission for Radiological Protection (ICRP), its primary aim is “to contribute to an appropriate level of protection for people and the environment against the detrimental effects of radiation exposure without unduly limiting the desirable human actions that may be associated with such exposure” (ICRP_103 2007). To this end, the system of radiological protection is based on three key principles:

- Justification: Any decision that alters the radiation exposure situation should do more good than harm.
- Optimisation of Protection: The likelihood of incurring exposure, the number of people exposed, and the magnitude of their individual doses should all be kept as low as reasonably achievable, taking into account economic and societal factors.
- Application of dose limits: The total dose to any individual from regulated sources in planned exposure situations other than medical exposure of patients should not exceed the appropriate limits specified by the Commission.

Radiation detriment is a fundamental concept of the ICRP system and is defined in the Glossary of ICRP Publication 103 as “A concept used to quantify the harmful health effects of radiation exposure in different parts of the body. It is defined by the Commission as a function of several factors, including incidence of radiation-related cancer or heritable effects, lethality of these conditions, quality of life, and years of life lost owing to these conditions” (ICRP_103 2007).

Notable is that although the aim of the Recommendations is to protect against the effects of radiation, the three key protection principles focus exclusively on exposure. This is because (weighted) absorbed dose is treated as the primary determinant of stochastic detriment within the operational system of radiological protection. The detriment varies for the non-modifiable factors sex, age at exposure and time-since-exposure/attained age (Grant et al. 2017) but is otherwise considered to be purely a function of dose and dose rate. The nominal level of cancer risk (defined by ICRP as sex-averaged and age-at-exposure-averaged lifetime risk estimate per unit dose for a representative population – ICRP 103) is derived from epidemiological studies, mainly the Life Span Study (LSS) of the Japanese atomic bomb survivors (Ozasa et al. 2019).

In practice, the strong coupling of dose and nominal risk can be interpreted by experts and non-experts alike as implying that cancer risk becomes fixed once exposure has occurred. This fatalistic attitude (understood here as the belief that events are predetermined by fate and that humans are powerless to change their destinies) is reflected in interviews of people potentially or actually exposed as a consequence of radiological emergencies (Zeleznik et al. 2020; Suzuki et al. 2015). Such an attitude is understandable because radiation protection estimates describe average risk in populations rather than the future outcome for any particular individual and risk is a concept that is difficult to define and understand (Schenk et al. 2019; Wojcik et al. 2018). Following Adams (2000), we distinguish between scientifically estimated (“actual”) risk and perceived risk. While estimates of actual risk are derived from epidemiological observations in populations, the way individuals perceive and interpret these estimates often differs substantially (Adams 2000; Perko 2014). Yet, the fatalistic attitude is unfounded because risk is inherently conditional and never an absolute, static number. Risk is fundamentally a measure of potential, uncertain outcomes that are conditioned upon specific scenarios or actions (Adams 2000; Aven 2010).

Important for protection against radiation-related detrimental stochastic effects is the realisation that, as outlined below, the risk of at least some radiation-related cancers can be modified after exposure by changing life-style factors. Such information should be passed to exposed and potentially exposed people because it can not only reduce the detriment but also potentially avert the feeling of fatalism which can severely impact mental and physical health (Carlson 2023). The aim of the present paper is to briefly review and summarise evidence for the conditionality of radiation-related detriment and suggest how appropriate recommendations for radiogenic cancer prevention should be designed and justified.

Because the present paper discusses a possible extension of the system of radiological protection through post-exposure lifestyle recommendations, it is appropriate to begin with the conceptual framework adopted in ICRP Publication 103 (ICRP_103 2007). Although Publication 103 is primarily concerned with the principles and application of radiological protection rather than providing a comprehensive review of radiation biology, its recommendations are founded on the contemporary understanding of radiation carcinogenesis and epidemiology. The following discussion, therefore, uses the ICRP framework as the point of departure and subsequently considers evidence from the broader and more recent mechanistic and epidemiological literature.

### How can changing the lifestyle prevent radiogenic cancer? Insights from mechanisms of carcinogenesis

ICRP Publication 103 (ICRP_103 2007) describes a conceptual framework in which radiation-related cancer may originate from DNA damage and misrepair events in a single stem-like (or stem-like behaving) target cell. Of particular importance for radiation-related cancer are complex forms of DNA double strand breaks that trigger DNA repair processes which, if not error-free, will lead to the formation of gene and chromosomal mutations. “Certain gene and chromosomal mutations, which are often tissue-specific, can confer cellular properties which allow these target stem cells to partially escape from their normal constraints of growth and development. In some cases, these cells acquire novel properties via gain of function mutations in so-called oncogenes; in others, it is loss of function of so-called tumour-suppressor genes that applies. On current hypotheses, the full potential for malignancy in these tumour-initiated cell clones is then developed in a step-wise fashion via the appearance of other gene/chromosomal mutations or in some cases the non-mutational silencing of key genes” (ICRP_103 2007). This model of carcinogenesis is based on the somatic mutation theory that dates back to 1914 when Theodor Boveri postulated that cancer arises in consequence of chromosomal rearrangements (reviewed in Brucher and Jamall 2016). Inherent to this theory is that cancer is derived from a single somatic cell that has accumulated multiple DNA mutations resulting in autonomous, uncontrolled proliferation free from normal constraints of growth and development exerted by the surrounding tissue (Alexandrov and Stratton 2014). Berenblum and colleagues developed in the 1940s the concept that carcinogenesis involves the distinct stages of initiation and promotion (reviewed in Shubik 2002). The concept of tumour progression was described later by Leslie Foulds, Harry Greene and Jacob Furth who recognised that neoplasms continue to evolve through successive qualitative changes towards increasing malignancy (reviewed in Sudilovsky et al. 1991). An initiator causes irreversible DNA damage and a promoter subsequently drives the expansion of that initiated cell. The concept was based on results of mouse skin painting experiments showing that cancer induction required the sequential application of two different types of carcinogens: an initiator followed by a promoter. Both agents are carcinogens, the former being a mutagen (for example 3,4-benzpyrene) and the latter a mitogen (for example phorbol ester). A question that followed was how many mutations are needed for a cancer to develop from a single cell. Based on the generalised observation that cancer mortality among individuals aged between 25 and 75 years developed proportionally to the sixth power of age, Nordling (1953) and Ashley (1969) suggested that the number is about 6. The two-stage theory of carcinogenesis developed by Armitage and Doll provided the mathematical formalism for the increase of cancer incidence with age (Armitage and Doll 1954; Ruhm et al. 2017). Inherent to the somatic mutation theory and the concept of initiation and promotion is the assumption that two carcinogens can only interact if one is an initiator and the other a promoter. A reflection of this idea can be found in ICRP 103, section A137 that discusses the mode of risk transfer between human cohorts (ICRP_103 2007).

In recent years, the validity of the somatic mutation theory has been questioned based on the argument that it fails to explain why, despite heterogeneous mutations, cancers of the same type and stage look identical (Huang et al. 2025). Many tumours have no identified driver mutations and normal tissues often harbour extensive cancer-driving mutations without becoming cancerous. Moreover, cancer cells implanted into normal tissue do not necessarily give rise to a tumour but may become normalised by the stroma. Hence, cancer does not necessarily arise from a linear accumulation of mutations in an initiating cell but is instead a dynamic process shaped by variation in cell plasticity, potential competition, cooperation, suppression and other clonal or cellular interactions within a complex tissue ecosystem (Brucher and Jamall 2016; Frank 2025; Fan 2026). From this perspective, the tumour microenvironment and stromal signals are not passive bystanders but seem to be active regulators of carcinogenesis because stromal constraints can suppress malignant behaviours whereas altered tissue organization can facilitate tumour initiation and progression (Wilczynski and Nowak 2022; Soto and Sonnenschein 2011). Proponents of the tissue organisation field theory of carcinogenesis go even so far as to claim that cancer is not a genetic disease starting from a single tumour-initiating cell but rather an anomaly of tissue organisation. This theory is based on the premise that under normal conditions, the propensity of normal cells to proliferate is blocked by tissue signals. Cancer arises when the tissue organisation constraint is weakened and cells are freed to express their default state, thus proliferating, generating variation and moving (Huang et al. 2025). In addition to the above, it is important to recall that radiation may also induce and promote carcinogenesis through epigenetic reprogramming, including DNA methylation changes, histone modifications and altered non-coding RNA expression, that can stably shift gene expression without changing the DNA sequence (Belli and Tabocchini 2020; Wei et al. 2019).

Different cancers may be driven predominantly by different combinations of genetic alterations and tissue-level constraints. The existence of two mechanisms of carcinogenesis (i.e., genetic and epigenetic) also explains why not all IARC class 1 carcinogens are mutagens (https://monographs.iarc.who.int/list-of-classifications). The fact that cancer development is a multi-step process that involves a multitude of changes in cells and tissues that influence each other provides a mechanistic explanation for how two carcinogens can interact potentiating the probability of cancer development. A logical conclusion is then that preventing exposure to one carcinogen can weaken the effect of the other. Here it is important to recall the key cancer surveillance function of the immune system (Kim et al. 2007; Corthay 2014). Transformed cells may be eliminated, enter equilibrium or eventually escape immune control as tumours acquire immune-evasive and inflammation-associated traits (Kim et al. 2007; Dunn et al. 2004). Lifestyle factors can weaken the immune system leading to enhanced risk of cancer development (Lanza et al. 2024). A prominent example of a lifestyle cancer promoter is cigarette smoke which attenuates the immune system’s ability to properly detect and control cancer cells (Jiang and Li 2024).

If carcinogenesis depends not only on initiating mutations but also on promotion/progression and immune surveillance, then interventions applied after exposure to radiation may still influence the probability of cancer emergence. The interesting question is what life-style factors can potentially enhance or weaken the carcinogenic effect of radiation? Ionising radiation can be regarded as a complete carcinogen in the sense that it can contribute both to initiation through mutagenic DNA damage and to promotion/progression through tissue injury responses including inflammation, compensatory proliferation and microenvironmental changes (BEIR_V 1990). It can also induce bystander and other non-targeted effects, meaning that non-irradiated neighbouring cells may exhibit DNA damage, chromosomal instability or carcinogenic responses after receiving stress signals from irradiated cells (Marin et al. 2015; Morgan and Sowa 2007). So, it can interact with any other carcinogens or factors that influence the immune system. According to a new assessment by the WHO, close to 40% of all cancers are attributable to modifiable risk factors, mainly tobacco smoking, alcohol consumption, high body mass index, insufficient physical activity and UV radiation (Fink et al. 2026). According to Barnard, smoking, a high-fat and refined-sugar diet along with a lack of physical activity represent the life-style factors that can be changed and may prevent the most common types of cancer like lung, breast, prostate and colorectal cancer (Barnard 2004). A similar conclusion was reached by Golemis et al. (2018). So, based on the above, it can be assumed that lifestyle modification can reduce not only the overall cancer risk in exposed populations but also, at least for some cancers, the fraction of risk attributable to radiation through interaction with promotion/progression pathways.

### Can lifestyle change prevent radiogenic cancer? Insights from epidemiology

ICRP Publication 103 acknowledges that if two human cohorts differ with respect to prevalence of modifiers of radiation-related cancer risk, their level of radiation-induced detriment will differ (ICRP_103 2007). This poses a challenge when transferring site-specific estimates of radiation-related risk (which is a process of taking data on cancer risk calculated for one group of people and applying it to a different group of people to predict their health outcomes) from one population to another if the corresponding cancer baseline rates differ (Wakeford 2012). Based on the two-stage initiation–promotion model (see preceding section for a description of the model), ICRP assumes that radiation acts primarily as a cancer initiator, so multiplicative transfer (assuming an excess rate that is directly proportional to the baseline rate) is plausible if the difference in cohort rates were associated with differential exposure to cancer promoters. On the other hand, additive transfer, when the excess rate is independent of the baseline rate, would be plausible if the rate difference could be ascribed to differential exposure to competing cancer initiators (ICRP_103 2007). As stated in the preceding section, ionising radiation and many other carcinogens are both inducers and promoters. Moreover, the simple two-stage initiation–promotion model is a simplification of how combined exposures work in real systems and interaction can occur whenever agents modify distinct or shared rate-limiting processes within multistage carcinogenesis (Lagunas-Rangel et al. 2022). Hence, predicting the effect (interaction or additivity) of sequential exposure to two carcinogens based on their mechanism of action is not straight forward.

The uncertainty regarding whether radiation-related cancer risk is proportional or additive to the baseline cancer rate is well reflected in documents of the US National Research Council Committee on the Biological Effects of Ionizing Radiations (BEIR). While BEIR III (BEIR_III 1980) assumed that the additive model applies to all cancer sites, BEIR V (BEIR_V 1990) assumed that multiplicative model applies to all sites. BEIR VII (BEIR_VII 2006) and ICRP 103 (ICRP_103 2007) assume that, with some exceptions, risk between cohorts should be transferred based on a weighted combination of additive and multiplicative models. A comparison of the percentage weightings assigned to the excess absolute risk (EAR) and excess relative risk (ERR) transfer models by ICRP 103 (and expanded upon by ICRP Publication 152) and BEIR VII is shown in Table 1. Breast cancer risks were based solely on an EAR model because a pooled analysis of eight cohorts of radiation-related breast cancer demonstrated a lack of interaction with base-line rate (Preston et al. 2002). In contrast, transfer of thyroid cancer risks was based solely on the ERR model developed from the pooled analysis of five cohorts of radiation-associated thyroid cancer (Ron et al. 1995). For transferring lung cancer risks, the ERR model was given a weight of 0.3 because of suggestions in the atomic bomb survivor data that the EAR is more comparable across sexes than the ERR, and also that radiation dose and smoking history interact additively as lung cancer risk factors (Pierce et al. 2003). For all other cancers, BEIR VII gives a greater weight to relative risk (0.7) than for absolute risk transfer between cohorts, whereas ICRP 103 gives equal weighting to relative and absolute transfer (Table 1). The reason for this is not clear and probably reflects a difference in expert judgment. Both committees agree that a substantial proportion of radiation-related cancers arise in proportion to baseline rates; consequently, the absolute number of radiation-attributable cases would be expected to vary with, and potentially be reduced by, lowering the baseline incidence.

**Table 1:** Percentage weighting assigned to the excess absolute risk (EAR) and excess relative risk (ERR) transfer models when transferring site-specific radiation-related cancer risks between populations with different baseline cancer rates, as recommended by ICRP Publications 103 (ICRP_103 2007) and Publication 152 (ICRP 2022) and BEIR VII (BEIR_VII 2006). The values indicate the percentage contribution of each transfer model to the final transferred risk estimate. Thus, a weighting of 100% indicates exclusive use of that model, whereas 0% indicates that the corresponding model is not used

Cancer type	ICRP Publication 103 and 152		BEIR VII	
	EAR weight	ERR weight	EAR weight	ERR weight
**Bone**	100	0	30	70
**Breast**	100	0	100	0
**Lung**	70	30	70	30
**Skin**	0	100	30	70
**Thyroid**	0	100	0	100
**All other**	50	50	30	70

Because radiation epidemiology is by necessity opportunistic with regard to the availability of data for testing how far radiogenic cancer is conditional, results providing evidence for the impact of lifestyle factors on the level of risk are scarce. Most data come from the Life Span Study of the Japanese survivors of the atomic bombings of Hiroshima and Nagasaki. A collection of studies known to us are highlighted below to provide an illustrative picture of the role lifestyle can play in modulating the risk of radiogenic cancer.

As mentioned above, the analysis of Pierce at al. (2003) suggested that smoking and ionising radiation do not interact (i.e., are additive) in producing lung cancer. More recent analyses of the LSS cohort do not support this and state that smoking potentiates the radiogenic lung cancer risk in a submultiplicative manner (i.e. smoking and radiation interact) (Furukawa et al. 2010; Cahoon et al. 2017). The same mode of interaction is observed following combined exposure to cigarette smoke and radon (Darby et al. 2005). A similar impact of cigarette smoking on lung cancer risk is seen among male and female members of the LSS cohort, suggesting that smoking potentiates the risk of radiogenic lung cancer even if began after radiation exposure. This conclusion is based on the fact that before World War II, smoking by young women in Japan was not socially acceptable and rare (Connolly et al. 2011). Restraint from or cessation of smoking can reduce the risk of radiogenic lung cancer. Land et al. (1994) showed that an early age at first full-term pregnancy, multiple births, and lengthy cumulative lactation protect LSS women against radiation-induced breast cancer. However, Shore et al. (1980) did not observe any potentiating effect of late parity on radiogenic breast cancer risk in LSS women. Hence, the impact of reproductive factors remains unclear. Several studies have been published demonstrating that a diet rich in fruit and yellow-green vegetables protects against radiation-related cancer of the bladder (Nagano et al. 2000), stomach, liver and lung (Sauvaget et al. 2003) and all cancers pooled (Sauvaget et al. 2004). The cancer risk reduction for multiplicative effect of diet was between 8 and 25%, depending on the cancer site. Sharp et al. (2003) found that hepatitis C but not hepatitis B infection potentiates the risk of radiogenic liver cancer among the LSS cohort members. In summary, several investigations of the LSS cohort demonstrate that radiogenic cancers can be prevented by changing the lifestyle. A similar conclusion was reached by Imaoka et al. (2016) for second primary cancers induced by radiotherapy and by authors of three studies on thyroid cancer induced by radiation (Zupunski et al. 2019; Pfinder et al. 2016; Shakhtarin et al. 2003).

It is relevant to note that for all types of solid cancer except thyroid cancer, following an exposure, ICRP models a decline of ERR with increasing attained age (ICRP 2022). This implies a reduction in the effects of the interaction of radiation with background risk factors with increasing time since exposure. Whether post-exposure lifestyle changes can alter this temporal evolution of ERR, and if so, by how much, remains unknown. This question remains open with potentially important implications for risk projection and post-exposure health guidance.

### Evidence from animal experiments that radiogenic cancer can be prevented

While epidemiological studies provide direct, real-world data on humans, they are observational and can be affected by various biases and are often confounded by multiple, simultaneous exposures. Animal experiments offer several critical advantages over human epidemiological studies, primarily stemming from the ability to rigorously control experimental conditions and to proactively assess safety before human exposure (Rall 1979). A number of animal studies (mainly using rodents) have been carried out to test the possibility of preventing radiogenic cancer by modifying lifestyle factors.

Animal studies have been conducted to test the impact of obesity and physical exercise on radiation-related cancer in rodents. The observations are summarised below. Cancer development induced by 3 Gy total body irradiation was higher in high fat diet-induced obesity and sedentary male as compared to lean mice of the CBA strain but attenuated by lifelong exercise after radiation exposure. The most common tumours were acute myeloid leukaemia and lymphocytic lymphoma in the spleen and bone marrow and hepatocellular adenoma and hepatocellular adenocarcinoma in the liver (Farber et al. 2021). A number of studies on mice and rats report that caloric restriction leads to a significant prolongation of cancer-free life span in the groups on a restricted diet after having been exposed to radiation, either before or after dietary restriction, in comparison with animals that were fed normal or fat-rich diet (Yoshida et al. 1997, 2006; Shang et al. 2014; Gross and Dreyfuss 1984, 1986; Silverman et al. 1980). The analysed cancer type was mostly leukaemia. Recently, Imaoka and colleagues analysed the impact of fat diet, parity, chemicals and birth cohorts on radiation-induced breast cancer in rats. They found that dietary fat exhibited a supramultiplicative interaction, chemicals represented a multiplicative interaction, and parity and birth cohorts displayed interactions that did not significantly depart from additivity or multiplicativity (Imaoka et al. 2023). Inano and Onoda reported that simultaneous administration of curcumin and diethylstilbestrol significantly reduces the development of mammary tumours in irradiated rats (2002). A recent review provided a mechanistic insight in the interaction of radiation and high fat diet. The authors found that high fat diets can exacerbate radiation-induced damage in cells and tissues by increasing oxidative stress, inflammation, and DNA damage, potentially elevating the risk of chronic diseases (Kantapan et al. 2025). Vares et al.(2014) found that high fat diet modulates radiation responses in mice, especially epigenetic regulations, possibly due to obesity-induced chronic oxidative stress.

Overall, these experiments suggest that radiation-related cancer outcomes in rodents can be modified by post-exposure changes in diet, body weight or physical activity, supporting results of epidemiological studies.

### What can be recommended and to whom?

The recommendations should start with stating that improving several lifestyle factors after radiation exposure can meaningfully reduce future cancer risk, even though it cannot erase past DNA damage. The guidance is intended for:

- Patients treated with radiation (e.g., radiotherapy or repeated diagnostic or interventional procedures).
- Workers occupationally exposed to radiation.
- People exposed in accidental or emergency settings (e.g., nuclear accidents or occupational exposure incidents).

Individuals that may particularly benefit from post-exposure health guidance are those experiencing anxiety or psychological distress after radiation exposure. The linear non-threshold (LNT) model used in radiological protection assumes lack of a “safe” radiation dose (Wojcik and Zolzer 2024), occasionally raising concerns about future cancer regardless of the magnitude of exposure. This may adversely affect well-being and quality of life. Recommendations should therefore be presented not only as measures with the potential to reduce risk of radiation-related cancer, but also as part of supportive risk communication that promotes understanding of probabilistic risk, counters fatalistic interpretations, and enhances a sense of personal agency. At the same time, it must be stressed that lifestyle changes can reduce but not eliminate the cancer risk. Also, lifestyle factors do not modify radiation-related cancer risk equally for all cancer sites.

Suggested, core lifestyle recommendations are:

A. Do not smoke; if you smoke - quit.

Why this matters: smoking is the strongest modifiable cancer risk factor. For lung cancer, smoking strongly amplifies radiation‑related risk (including medical radiation to the chest and radon exposure). Evidence shows that stopping smoking reduces future lung cancer risk even after past radiation exposure. Recommendation: avoid smoking and second‑hand smoke completely. Smoking cessation should be considered the highest‑priority intervention after radiation exposure.

B. Try to maintain a healthy body weight (avoid obesity).

Why this matters: excess body fat increases the risk of several cancers through inflammation, insulin resistance, and growth‑factor signalling. These same pathways are relevant to cancer promotion and progression. Recommendation: aim for gradual, sustainable weight management through diet and physical activity. Avoid framing weight loss as urgent or extreme; long‑term metabolic health is the goal.

C. Be physically active and reduce sedentary time.

Why this matters: regular physical activity reduces overall cancer risk and improves metabolic and immune health. Physical activity supports weight control and reduces chronic inflammation. Survivorship guidelines consistently recommend exercise to reduce risk of new cancers and improve long‑term outcomes (Rock et al. 2022). Recommendation: aim for regular aerobic activity plus muscle‑strengthening exercises, adapted to individual capacity. Any increase in activity is beneficial; perfection is not required.

D. Limit alcohol consumption.

Why this matters: alcohol increases the risk of several cancers. Recommendation: if alcohol is consumed, keep intake low. Avoid alcohol entirely if possible, especially for individuals with other cancer risk factors (such as obesity or genetic predisposition).

E. Support overall health (sleep, stress, medical follow‑up).

Why this matters: adequate sleep, stress management, and routine healthcare support metabolic and immune health. While not proven to specifically reduce radiation‑attributable cancer risk, these factors improve overall resilience and quality of life. Recommendation: maintain regular sleep patterns. Seek support for chronic stress. Participate in age‑ and sex‑appropriate cancer screening and medical follow‑up.

Depending on the exposure situation and the local possibilities, the recommendations should be accompanied by offer of institutional support such as healthy workplace conditions, access to cessation programs and community resources.

### Areas outside of radiological protection where change of lifestyle is recommended to prevent detriment

Lifestyle and behavioural recommendations as a component of post-exposure health protection are well established in several domains outside radiological protection. In environmental and chemical risk management, public health agencies routinely complement exposure reduction with guidance on modifiable behaviours, for example dietary recommendations to reduce lead absorption (e.g., adequate calcium and iron intake) and food-consumption advisories to reduce mercury exposure from fish while maintaining nutritional benefits (https://www.efsa.europa.eu/en/news/fish-and-seafood-consumption-eu-awareness-dietary-advice-mercury). Similarly, occupational health guidance for asbestos-exposed populations emphasizes smoking cessation, reflecting the well-known combined effects of smoking and inhaled carcinogens on lung cancer risk (DASAM 2013). In medicine, lifestyle modification is widely integrated into standard care pathways after diagnosis or intervention, such as physical activity and weight management in diabetes prevention and management, and structured secondary prevention through cardiac rehabilitation after myocardial infarction (Brinks et al. 2017). Importantly, compliance with lifestyle recommendations is variable and depends strongly on supportive program design. For example, participation and retention in cardiac rehabilitation remain suboptimal and retention in structured diabetes prevention programs is incomplete (Jin et al. 2008). These precedents support the argument that evidence-based lifestyle recommendations can legitimately complement radiological protection after radiation exposure, provided they are framed as supportive and empowering rather than as substitutes for exposure prevention.

### Ethical and philosophical considerations

Recommendations for lifestyle modification following radiation exposure raise ethical questions concerning autonomy, responsibility and the extent to which individuals can reasonably be asked to reduce their own health risks. Importantly, the proposal to encourage healthy behaviour should not be interpreted as shifting responsibility for radiation exposure from institutions to individuals. Primary prevention through exposure avoidance and dose reduction remains the central obligation of the system of radiological protection and lifestyle recommendations must be framed explicitly as complementary rather than substitutive measures.

From a philosophical perspective, such recommendations may also be challenged by deterministic interpretations of human behaviour, which could be misperceived as implying that individual actions cannot influence future outcomes (Dennett and Caruso 2021). However, determinism does not imply fatalism: even in a causally determined framework, behavioural choices and environmental conditions remain part of the causal chain influencing future health outcomes. Therefore, providing individuals with evidence-based information on modifiable risk factors can be ethically justified as a means of supporting agency and enabling informed decision-making, rather than as a moral judgement.

Importantly, ethical implementation requires attention to fairness and feasibility. Individuals differ substantially in their capacity to adopt lifestyle changes due to socioeconomic constraints, occupational demands, co-morbidities, psychological stress or limited access to preventive healthcare. Lifestyle guidance should therefore be communicated in a non-stigmatizing manner and accompanied, where possible, by institutional support measures (e.g., smoking cessation programs, occupational health services and accessible medical follow-up). Under these conditions, lifestyle recommendations can be understood as an empowering component of post-exposure health guidance that supports individuals in maintaining a sense of control and dignity without undermining established principles of radiological protection. It should be noted that Ohtsura Niwa has repeatedly emphasised that the risk of radiogenic cancer is influenced by lifestyle and environmental factors and that lifestyle recommendations can help affected people to regain control over their situation and restore dignity after a nuclear accident (Niwa 2023). Experiences from the Fukushima dialogue process indicate that the development of a practical culture of radiological protection can help people regain dignity and confidence in shaping their future after a nuclear accident (Lochard et al. 2019).

Beyond individual health benefits, lifestyle recommendations may also be viewed as supporting societal resilience in the sense of an “open society,” as described by Karl Popper (Popper 2011). In this perspective, radiological protection is not only a technical system of dose assessment and regulation but also a social practice based on transparency, critical discussion and informed decision-making, much in line with recommendation of ICRP 138 (ICRP_138 2018). Providing affected individuals with evidence-based guidance on modifiable risk factors can therefore contribute to empowerment and reduction of fatalistic attitudes, while reinforcing trust through honest communication of uncertainties and limitations. Such an approach is consistent with participatory models of risk governance, in which institutions retain responsibility for exposure prevention while citizens are supported in taking practical actions to prevent detriment (Hedelin et al. 2017).

### Possible misinterpretations of the recommendations

It can be expected that attempts to implement lifestyle recommendations for exposed people will be met with scepticism due to misinterpretation of their aims. The main points of criticism can be summarized as follows: (1) responsibilisation of citizens; (2) political misuse to weaken protection standards; (3) going beyond the remit of radiological protection; (4) problem of equity. The points and suggested responses are shortly discussed below.

1. Responsibilisation of citizens. The recommendations can be viewed as an attempt to shift responsibility from institutions (industry, regulators, governments) to individuals. Radiation exposure is often involuntary; lifestyle advice implies that the involved person is responsible for his/her own cancer risk. The recommendations can be seen as “healthism”: moralising health behaviours (Brown 2018). The response is that lifestyle recommendations do not replace the duty to prevent exposure; they are an additional, supportive option for people who want to reduce overall risk and regain control of their fate.
2. Political misuse to weaken protection standards. Representatives of industry or governmental organisations may be willing to weaponize lifestyle messaging by claiming that radiation is not the problem – obesity (or another lifestyle factor) is. In this way, the recommendations become a tool for minimizing accountability. An example can be found in the fossil fuel industry (Supran and Oreskes 2021). In this way it may support actions to weaken protection standards (https://thebulletin.org/2025/10/president-trumps-radical-attack-on-radiation-safety). The response is a clear statement that lifestyle advice must not be used to replace or weaken radiation protection standards. Obviously, lifestyle recommendations may not be used to justify higher exposure limits. However, the argument that recommendations that prevent detriment should be withheld because they can be misused do not conform to the ethical principle of beneficence (ICRP_138 2018).
3. Going beyond the remit of radiological protection. The claim may be expressed that radiological protection is about dose limitation, not lifestyle coaching and that lifestyle recommendations belong to the medicine/public health territory, diluting the discipline and confusing responsibilities. The response is that the primary aim of radiological protection is to protect against detriment, not exposure. Lifestyle recommendations do not represent medical interventions but volunteer actions to be taken by exposed people to prevent harm. In this way, the recommendations complement the system moving beyond the limitations of exposure prevention.
4. Problem of equity: people cannot always change lifestyle. The argument may be used that lifestyle recommendations are unfair and exert moral pressure on exposed people who may have limited control over diet, exercise, sleep due to shift work or may not have access to healthy food or healthcare. The response is that the recommendations should be accompanied by institutional support such as healthy workplace conditions, access to cessation programs and community resources. But to echo the statement made in point 2 above: the argument that recommendations should be withheld due to lack of equal opportunities goes against the ethical principle of beneficence (ICRP_138 2018).

## Conclusions

Lifestyle advice is not a substitute for radiation protection standards; it is an additional, empowering health measure that can reduce overall cancer risk and may reduce progression of radiation-initiated lesions. Even if some determinants of health behaviour lie beyond individual control, lifestyle interventions remain ethically justified as supportive measures because they provide practical options for detriment reduction. They may also serve as an empowering component of post-exposure health guidance by providing practical actions that help restore a sense of control and dignity. Such recommendations should not be framed as moral obligation or blame, but as empowerment, and should be accompanied by societal and institutional support.

## Data Availability

No datasets were generated or analysed during the current study.
